
JOURNAL INFORMATION
==============================
NLM Title Abbreviation: ScientificWorldJournal
Journal ID: ScientificWorldJournal
NLM Journal ID: 101131163
Journal ID: TSWJ

The Scientific World Journal

ISSN: 2356-6140
EISSN: 1537-744X
Publisher: Wiley

ARTICLE INFORMATION
==============================
PMCID: PMC6083895
PMID: 30147515
DOI: 10.1100/tsw.2001.191
Article ID: 209326
Article version: 1
Subjects: Short Report

Selective Effect of Ethanol on Mouse Blastocyst Development

Hsuuw Y-D 1 *
Lee K-H 2
Kuo T-Fu 3
1 Department of Nutrition and Health Science, Fooyin Institute of Technology, Taiwan
2 Department of Applied Biology, Pig Research Institute Taiwan, Taiwan
3 Department of Veterinary Medicine, National Taiwan University, Taiwan
*Y-D Hsuuw: hsuuw@kimo.com.tw
Publication date: 2001
Electronic publication date: 2001 Nov 27
Volume: 1
First page: 53
Last page: 53
Copyright: Copyright © 2001 Y-D Hsuuw et al.
Copyright year: 2001
License: This is an open access article distributed under the Creative Commons Attribution License, which permits unrestricted use, distribution, and reproduction in any medium, provided the original work is properly cited.
License URL: https://creativecommons.org/licenses/by/3.0/

==============================
Miami *Nature Biotechnology* Short Reports
*TheScientificWorld* (2001) 1 (S3), 53SR
ISSN 1532-2246; DOI 10.1100/tsw.2001.191

### SELECTIVE EFFECT OF ETHANOL ON MOUSE BLASTOCYST DEVELOPMENT

Y-D Hsuuw_*(1), K-H Lee (2), and T-Fu Kuo (3)

(1) Department of Nutrition and Health Science, Fooyin Institute of Technology, Taiwan; (2) Department of Applied Biology, Pig Research Institute Taiwan, Taiwan; (3) Department of Veterinary Medicine, National Taiwan University, Taiwan
* Hsuuw@kimo.com.tw

**INTRODUCTION.** The clinical evidence has revealed that moderate or chronic ingestion of alcohol is involved in the embryopathy, reproductive failure, and fetal malformation (1). The administration of alcohol in vivo resulted in inhibiting of the ovulation, fertilization (2,3).

**METHODS.** Blastocysts were obtained from day 4 of pregnancy. Embryos were cultured in the presence of ethanol (0.1, 0.2 and 0.3%) for 24 hours. Morphological score were observed according to the cavity expand and the zona pellucida exclusion of blastocysts. Blastocysts were examined by dual differential staining, which allow to realizing the population of ICM and TE cells. Cells containing the fragmented nuclei were identified as apoptotic cell, and cells with disrupted membrane were considered as necrotic cells.

**RESULTS.** Mouse blastocysts with medium stage were respectively cultured in different concentration of ethanol. Results showed that ethanol exposure had a significantly delay on embryo development researching to the hatched stage ($P \leq 0.05$). This inhibitory effect of ethanol on embryo development were further determined by using a dual staining method, which allow to realize the both cell lineages of blastocysts, inner cell mass (ICM) and trophectoderm (TE). Blastocysts cultured in the dose of 0.2% or 0.3% ethanol treatment showed a dose-dependent decrease in their total cell number (Table 1, $P \leq 0.05$). Analysis of the two cell lineages of blastocyst, we found that ethanol decrease the cell population of ICM cells without affecting on the TE cells (Table 1, $P \leq 0.05$). Embryonic cell containing the fragmented nuclei was identified as apoptosis, and cell stained with trypan blue was considered as necrosis. The frequency of apoptotic or necrotic cell per embryo was not increased follow ethanol treatment.

**Table 1. Effect of ethanol on mouse blastocyst development in vitro**

| | Control | 0.2 % | 0.3 % |
|---|---|---|---|
| Total cell number | 117.07 ± 2.88 | 108.5 ± 2.47* | 106.85 ± 4.57* |
| ICM cells | 23.67 ± 1.74 | 16.89 ± 1.61* | 15.65 ± 1.62* |
| TE cells | 93.46 ± 2.83 | 91.58 ± 2.89 | 91.20 ± 4.15 |

*$P \leq 0.05$ between ethanol treatment and control embryos

**DISCUSSION.** Most of studies about the effect of alcohol on preimplantation embryo development are based on the morphological observation of those embryos. In our studies, blastocysts were exposed to the ethanol at the ranges from 0.1 to 0.3% in culture medium. We found that high dose of ethanol exerts a negative effect on embryo development. This ethanol-induced negative effect on cell growth of blastocysts is mediated by ethanol selective inhibiting the cell population of ICM cells without affecting on the TE cells. Previous reports have showed that zygotes exposed to a lower dose of ethanol (0.1%) in minutes results in promoting the blastocyst formation and further attachment. The enhancement of ethanol on embryo development is associated with the elevations of c-myc expression (1) and intracellular calcium ions (2-3). However, the detrimental actions of ethanol on preimplantation embryos are still unknown. Our results also indicated that the selective inhibition of ethanol on ICM cell growth was not mediated with cell killing by ethanol. This implied that the detriment of ethanol on postimplantation development might involve in the disruption of embryonic cell differentiation.

**REFERENCES.**

1. Leach, R.E., Rout, U.K., Schultz, J.F., Saunders, D.E., and Armant, D.R. (1999) Alcohol Clin. Exp. Res. 23, 778-784
2. Stachecki, J.J., Yelian, F.D., Leach, R.E., Armant, D.R. (1994) J. Reprod. Fertil. 101, 611-617
3. Stachecki, J.J., Yelian, F.D., Schultz, J.F., Leach, R.E., Armant, D.R. (1994) Biol. Reprod. 50, 1-9
